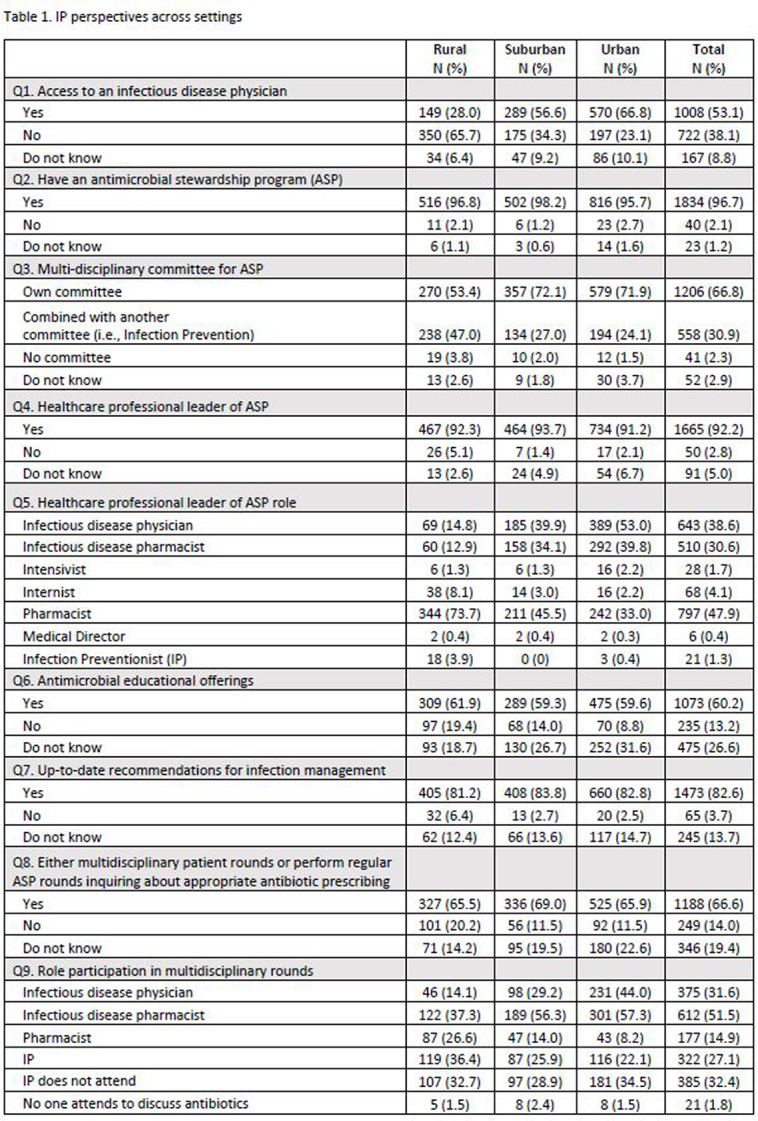# 92 Beyond interviews: Using visual methods to understand patient experiences with in-home IV antibiotic therapy and IV-line care

**DOI:** 10.1017/ash.2026.10516

**Published:** 2026-06-23

**Authors:** Elizabeth Monsees, Sara Reese, Lisa Hall, Annie Wirtz, Monika Pogorzelska-Maziarz

**Affiliations:** 1 Children’s Mercy Hospital; 2 APIC; 3 The University of Queensland; 4 Children’s Mercy Kansas City

## Abstract

**Background:** Since 2014, the Centers for Disease Control and Prevention’s Core Elements of Hospital Antibiotic Stewardship Programs have evolved to incorporate multidisciplinary practices that optimize therapy and minimize antibiotic-associated harm. Infection preventionists (IPs) play a key role in stewardship efforts, especially education and reporting of event data; this study examined IP perceptions of inpatient stewardship programs. **Methods:** Members and non-members of the Association for Professionals in Infection Prevention (APIC) were invited to complete the quinquennial electronic survey between June and July 2025 through numerous emails, QR codes at the annual APIC conference, and social media campaigns. The survey assessed nine antimicrobial stewardship program (ASP) characteristics by practice setting (rural, suburban, urban), including structure, leadership, and educational resources. Descriptive statistics were used to summarize the data. **Result:** Responses were received from 1898 IPs in acute care hospitals. Most (n=1834, 96.7%) reported the presence of an ASP (Table 1). Only 5% (n=91) were unaware of program leadership, less often in rural settings (n=13, 2.6%) compared with 6.7% (n=54) in urban settings. Rural settings (n=238, 47.0%) were more likely to combine the antibiotic stewardship committee setting with other committees, such as an IP committee, compared to urban settings (n=194, 24.1%). Clinical pharmacists (n=797, 47.9%) and infectious diseases physicians (n=643, 38.6%) were most frequently identified as leaders, while IPs represented 1.3% of ASP leaders (n=21). Urban (n=570, 66.8%) and suburban (n=289, 56.6%) settings had greater access to infectious diseases physicians compared to rural settings (n=149, 28%). Over 80% (82.6%, n=1473) reported the use of up-to-date recommendations for infection management, and education was reported by 60.2% (n=1073), though 26.6% (n=475) were unsure if antibiotic prescribing education was specifically offered – findings consistent across settings. Multidisciplinary ASP rounding occurred in 66.6% (n=1188) of hospitals regardless of setting, with IPs attending in 27.1% (n=322) of institutions. IPs (n=119, 36.4%) were more likely to attend rounds in rural settings. **Conclusion:** ASPs are well-established in acute care settings, typically led by pharmacists and physicians. However, IPs are less frequently engaged in ASP activities, with limited participation in rounding and awareness of educational initiatives. An exception exists in rural settings, where IPs assume broader stewardship responsibilities. Expanding IP involvement and enhancing educational outreach across all care environments represent key opportunities to strengthen stewardship and improve patient outcomes.

**Figure:**